# Effect of Chemical Disinfection on Chitosan Coated PMMA and PETG Surfaces—An In Vitro Study

**DOI:** 10.3390/polym10050536

**Published:** 2018-05-16

**Authors:** Katarzyna Walczak, Jessica Thiele, Daniel Geisler, Klaus Boening, Mieszko Wieckiewicz

**Affiliations:** 1 Department of Prosthetic Dentistry, Faculty of Medicine Carl Gustav Carus, Technische Universität Dresden, Fetscherstr. 74, 01307 Dresden, Germany; thiele.jessica@gmail.com (J.T.); Klaus.Boening@uniklinikum-dresden.de (K.B.); 2 Division of Psychological and Social Medicine and Developmental Neuroscience, Faculty of Medicine Carl Gustav Carus, Technische Universität Dresden, Fetscherstr. 74, 01307 Dresden, Germany; Daniel.Geisler@uniklinikum-dresden.de; 3 Department of Experimental Dentistry, Faculty of Dentistry, Wroclaw Medical University, 26 Krakowska st., 50-425 Wroclaw, Poland; m.wieckiewicz@onet.pl

**Keywords:** chitosan, chitosan acetate, surface, disinfection, PMMA, PETG

## Abstract

In oral sciences, chitosan application is of interest due to its antimicrobial and hemostatic activity. Chitosan coating of dentures and other intraoral devices could be beneficial for treatment of denture stomatitis or in the management of postoperative bleeding. Disinfection of dentures and prosthodontic materials is crucial before their use in patients. This study investigated the influence of chemical disinfectants on chitosan-coated surfaces. A total of 100 specimens were made: 50 of PMMA (polymethyl methacrylate), and 50 of PETG (polyethylene terephthalate glycol-modified) material and coated with 2% chitosan acetate solution. In each material, 5 groups (10 specimens each) were established and disinfected with Printosept-ID (L1), MD 520 (L2), Silosept (L3), or Dentavon (L4), or stored in distilled water (L0, control group). After disinfection, all specimens underwent abrasion tests (30,000 cycles in a tooth-brushing simulator). Areas without chitosan coating were measured by digital planimetry both before and after the disinfection/abrasion procedure and a damage-score was calculated. Regarding chitosan coating, the statistical analysis showed a significant influence of the disinfectants tested and significant differences between disinfectants (*p* < 0.05). Chitosan coating was most stable on PMMA and PETG after disinfection with MD 520 (L2). Otherwise, active oxygen containing disinfectants (L3, L4) led to the greatest alterations in the chitosan coating.

## 1. Introduction

**Figure 1:**
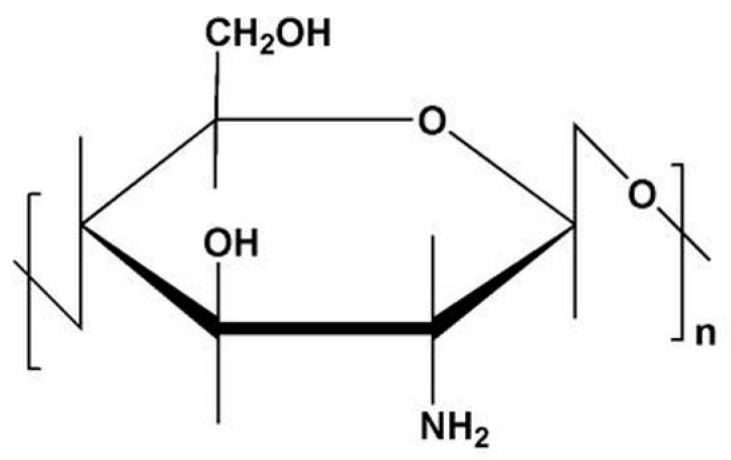
Chemical structure of chitosan (CS).

The biopolymer chitosan (CS, Figure 1) [a (1→4) 2-amino-2-deoxy-β-d-glucan] is a semi-synthetic aminopolysaccharide derived from chitin by *N*-deacetylation [1,2,3]. In nature, chitin is, after cellulose, the second most common biopolymer and it is synthetized by a wide range of species, for example crustaceans, insects and fungi [1,2,4]. CS is a linear polymer with reactive amino and hydroxyl groups [5]. In recent decades, CS has been of interest for many applications as a biomaterial [1,2]. CS is known to be non-toxic, biocompatible and biodegradable; it shows a range of beneficial biological activities, among others, as an antimicrobial and hemostatic agent, in wound healing or tissue engineering [1,5,6,7,8,9,10,11,12].

The properties and activities of CS depend on many factors, for example, molecular weight (MW), degree of polymerization (DP), degree of deacetylation (DDA), environmental effects, and so on [12]. The MW of CS can be categorized as follows: high (HMW > 300 kDa), medium (MMW > 190–300 kDa), low (LMW > 16–190 kDA) and oligo-CS ≤ 16 kDa [12].

Different applications of forms of CS have been studied, for example as hydrogels, membranes (films), fibers, sponges, microspheres, and capsules [1,13,14]. Also, coating procedures have been described [15].

Because of its regenerative and antimicrobial activities, CS is a topic of research in dentistry [4,16,17,18]. Moreover, CS is already used as a component in toothpastes, mouth rinses and dental dressings [16,18]. To develop new approaches to bleeding control management or the treatment of oral mucosal infections, new ideas for the using CS in dentistry have arisen and these have been discussed in our previous studies [19,20].

**Figure 2:**
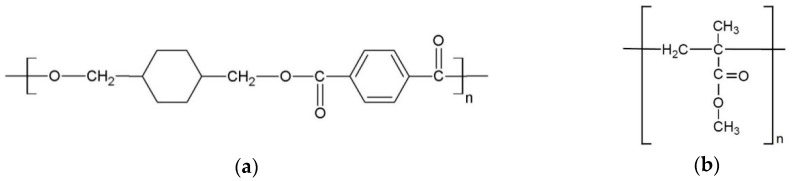
Chemical structure of (**a**) polyethylene terephthalate glycol-modified (PETG); and (**b**) polymethyl methacrylate (PMMA).

Due to demographic changes, there is an increasing number of elderly people in our society. Elderly people often have 5 to 10 remaining teeth and wear removable partial or if edentulous total dentures [21]. Removable dentures can induce denture stomatitis, a common oral mucosal infection among denture wearers with a prevalence of up to 70%. The etiology of denture stomatitis is unclear, but it is associated with colonization of the dentures with *Candida albicans* [22]. Prevention of the attachment and growth of microorganisms on dentures is one of the important factors in the treatment of denture stomatitis. [22]. Also, application of intraoral splints after surgical treatment in the oral cavity is a well-known method to compress the treated tissue and minimize bleeding [23]. For this purpose, individually prepared surgical splints or existing dentures can be used. Such surgical splints are commonly made from PETG (Figure 2a) or cold cured acrylic (PMMA, Figure 2b), and dentures of heat or cold cured acrylic (PMMA).

Our previous studies introduced a method for the surface coating of surgical splints and dentures with CS [19,20]. This method enables the abrasion resistant coating of PMMA and PETG materials with CS. Our previous studies showed that a long-term abrasion stable CS coating can be achieved by silicatization of PMMA and PETG material surfaces, subsequent coating with CS acetic solution and neutralization with NaOH [19,20]. Due to their excellent CS activities and properties, CS coated dental devices can be an alternative to standard therapy for oral diseases and surgical complications such as hemorrhage, and denture stomatitis.

**Figure 3:**
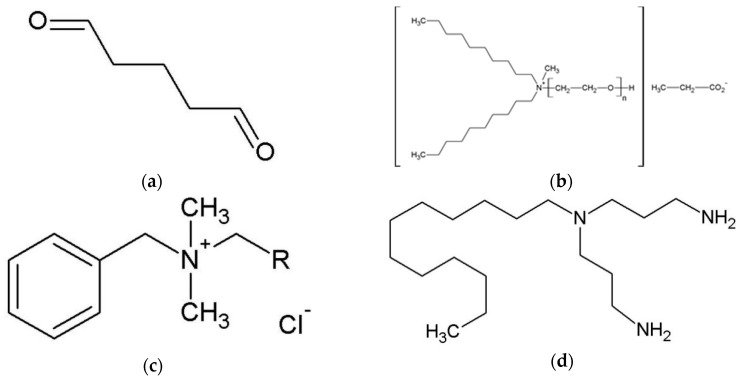

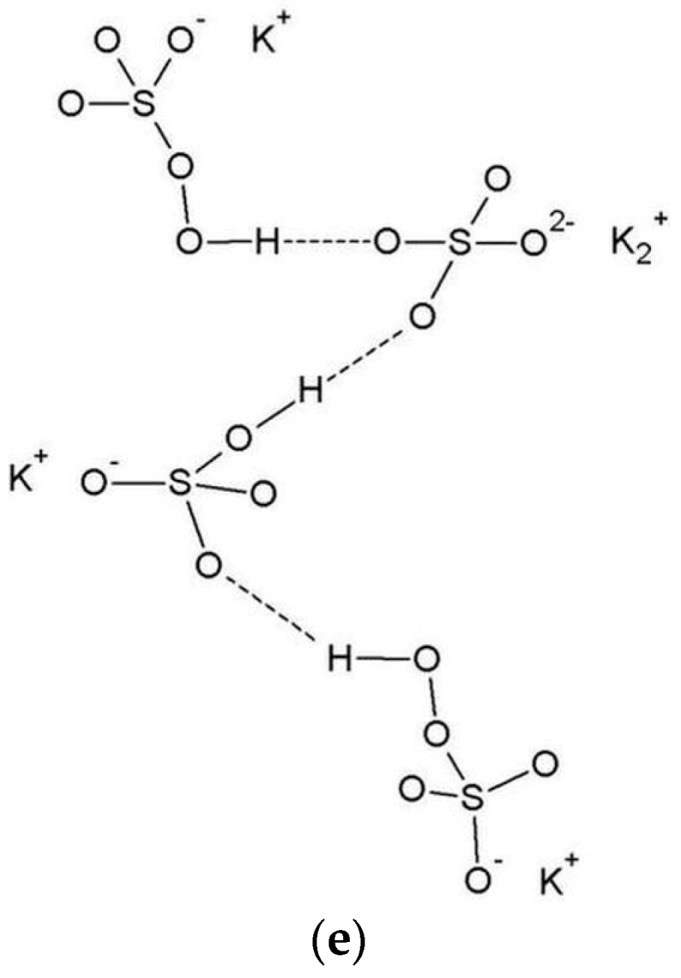
Chemical structure of (**a**) glutaraldehyde (GA); (**b,c**) quaternary ammonium compounds (QUATs); (**d)** alkyl amine; and (**e**) pentapotassium bis(peroxymonosulfate) bis(sulfate) (MPS).

Surgical splints and dentures are commonly prepared in a dental laboratory. To minimize the risk of cross contamination all dentures, and prosthodontic materials prepared in a dental laboratory or transported between a laboratory and a dental practice, have to be sterilized or disinfected [24,25,26]. Steam sterilization in an autoclave is a common method for medical/dental devices, but it is limited to heat and moisture resistant materials. For all other materials, for example polymers, thermal degradation, decompensation and hydrolysis can be induced [27]. Especially due to the low thermal stability of thermoplastic materials such as PMMA and PETG and the low ebullition temperature of the monomers in the PMMA, steam sterilization is not recommended for dentures or surgical splints [28]. Accordingly, application of chemical disinfectants is an internationally accepted and recommended method for decontamination of dentures and prosthodontic materials [25,26]. The following minimal requirements are given: bactericidal, levurozide, and limited virucidal activity or minimum intermediate-level of disinfection [25,26]. In Europe, currently available commercially manufactured chemical disinfectants for dentures and prosthodontic materials contain for example glutaraldehyde (GA), quaternary ammonium compounds (QUATs), alkyl amine or active oxygen (Figure 3a–e).

Glutaraldehyde (GA, Figure 3a) is a broadly used and studied high-level disinfectant [28,29,30,31]. GA is a water-soluble, amine-reactive, protein cross-linker with two aldehyde groups. The biocidal activity of GA is promoted through aldehyde groups due to alkylation reactions of sulfhydryl, hydroxyl, carboxyl, and amino groups in microorganisms [31]. This reaction leads to alteration of RNA, DNA, and protein synthesis [31]. GA is not corrosive and does not strongly influence the properties of acrylic or rubber materials, but has high toxic potential [28,31,32,33]. GA can cause skin or mucous membrane irritation [31]. During the disinfection process of dentures or other acrylic appliances, GA can penetrate into the acrylic resin surface and later cause allergies or inflammations [34].

Quaternary ammonium compounds (QUATs) are also widely used as disinfectants [31]. QUATs are surface-active and water-soluble; they act bactericidally through the inactivation of energy-producing enzymes, denaturation of essential cell proteins, and disruption of the cell membrane [31]. QUATs interact ionically with phospholipids in microorganism membranes and impair membrane permeability [35,36]. *N*,*N*-Didecyl-N-methylpoly(oxyethyl)ammonium propionate (Figure 3b) and alkyl-benzyl-dimethyl-ammoniumchloride (Figure 3c) are examples of QUATs.

Alkyl amines such as *N*-(3-aminopropyl)-N-dodecylpropane-1,3-diamine (Figure 3d) belong to the non-QUATs cationic tensides and are also widely used as compounds in QUAT disinfectants [37].

Active oxygen such as pentapotassium bis(peroxymonosulfate) bis(sulfate) (MPS, Figure 3e) is a high-level, water-soluble and biodegradable disinfectant [38]. This oxidizing agent can produce hydroxyl as well sulfate radicals, which can induce selective oxidation [39]. Generally, oxidation can affect thiol groups in microorganisms, whereupon hydroxyl radicals attack membrane lipids, DNA and other essential cell components [31,36].

Thus, the disinfection of dental prostheses, and prosthodontic materials with chemical disinfectants is recommended before use. Effective, non-destructive methods for CS coating disinfection are of high importance and have to be investigated. Otherwise, the use of CS coated dentures or surgical splints is not feasible for patients.

The aim of this study was to evaluate, using an abrasion test, the influence of commonly used chemical disinfectants on CS coated surfaces after ageing. The following null hypotheses were stated:

- Chemical disinfectants have no influence on CS coating abrasion resistance.
- There are no differences between the tested chemical disinfectants regarding abrasion resistance of CS coatings.
- There are no differences between PMMA and PETG materials regarding the abrasion resistance of CS coatings.

## 2. Materials and Methods

### 2.1. Establishing Specimens

Cylindrical PMMA (*n* = 50) and PETG specimens (*n* = 50) with a diameter of 12.75 mm were produced and coated with CS according to the protocol published in our previous study [20]. Ten test groups with 10 specimens each were established. One hundred PMMA (Palapress, Kulzer, Hanau, Germany) specimens were prepared according to the manufacturer’s instructions. Ten grams of PMMA powder was mixed with 7 mL of monomer liquid for 15 s at room temperature (23 °C), poured into a casting mold and polymerized for 20 min at 55 °C under 2.5 bar pressure. After polymerization, the samples were smoothed with 1000 grit sandpaper. To 50 of the PMMA samples PETG disks of Erkodur clear (Ø 12.75 mm, 2.0 mm thick, ERKODENT Erich Kopp GmbH, Pfalzgrafenweiler, Germany) were glued (cyanoacrylate glue, Renfert GmbH, Hilzingen, Germany). Chitosan 90/500 (Chitoscience, DDA 87.6–92.5%, MW 200–400 kDa, Heppe Medical Chitosan GmbH, Halle, Germany) was used for the coating solution. To prepare the 2% CS acetate solution, the CS was dissolved in 2% acetic acid (UKD Pharmacy, Dresden, Germany) using a magnetic stirrer (RET CV S000, IKA, Staufen, Germany) at a temperature of 60 °C. All specimens were sandblasted with Rocatec Pre (2.8 bar, 110 µm, 20 s, 3 M, Seefeld, Germany), silicatized with Rocatec Plus (2.8 bar, 110 µm, 20 s, 3 M, Seefeld, Germany) and air blasted for cleaning. The PMMA and PETG specimens were replaced in the casting mold and coated with 1 mm CS acetate solution followed by drying for 120 min at 45 °C in an incubator (B6030, Heraeus, Hanau, Germany). All specimens were neutralized in 1 mol/L NaOH solution for 10 min and afterwards washed in distilled water.

### 2.2. Disinfectants

Four chemical disinfectants commonly used and verified for disinfection of dentures and prosthodontic materials were used in this study: Printosept-ID, MD 520, Silosept, and Dentavon (hereafter, L1, L2, L3 and L4, respectively) (Table 1). All disinfectants were prepared and used according to directions for use (Table A1, Appendix A).

### 2.3. Measurement of Damaged Chitosan Coating Area (DCSCA)

**Figure 4:**
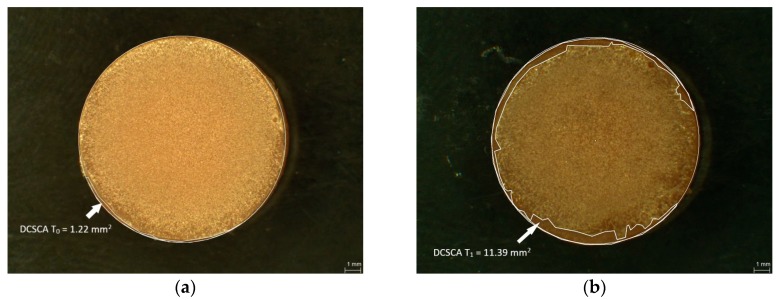
Selected specimens showing measurement of damaged chitosan coating area (DCSCA) at baseline T_0_, and T_1_ (after disinfection/abrasion test): L2 PMMA specimen DCSCA at T_0_ (**a**); L2 PMMA specimen DCSCA at T_1_ (**b**).

The CS coatings of the PMMA and PETG specimens were examined under a light microscope by digital planimetry (magnification 31.5×, Leica MZ12, Meyer Instruments, Houston, TX, USA). The first measurement of the damaged chitosan coating area (DCSCA) was conducted directly after the coating procedure (baseline, T_0_) and the second (T_1_) after disinfection followed by the abrasion test (Figure 4a,b). DCSCA was defined as the specimen surface without chitosan coating and was measured in mm^2^.

### 2.4. Disinfection Procedure and Abrasion Test

CS-coated PMMA (*n* = 40) and PETG (*n* = 40) specimens were immersed in four different chemical disinfectants (*n* = 10 per each) for a time-period according to the manufacturer’s directions for use: 5 min in disinfectant L1 and 10 min in disinfectants L2, L3, and L4 (Table A1, Appendix A).

After the disinfection procedure, the specimens were rinsed with tap water and air blasted. The specimens in the control group (L0, PMMA: *n* = 10, PETG: *n* = 10) were immersed in distilled water for 5 min and air blasted afterwards.

**Figure 5:**
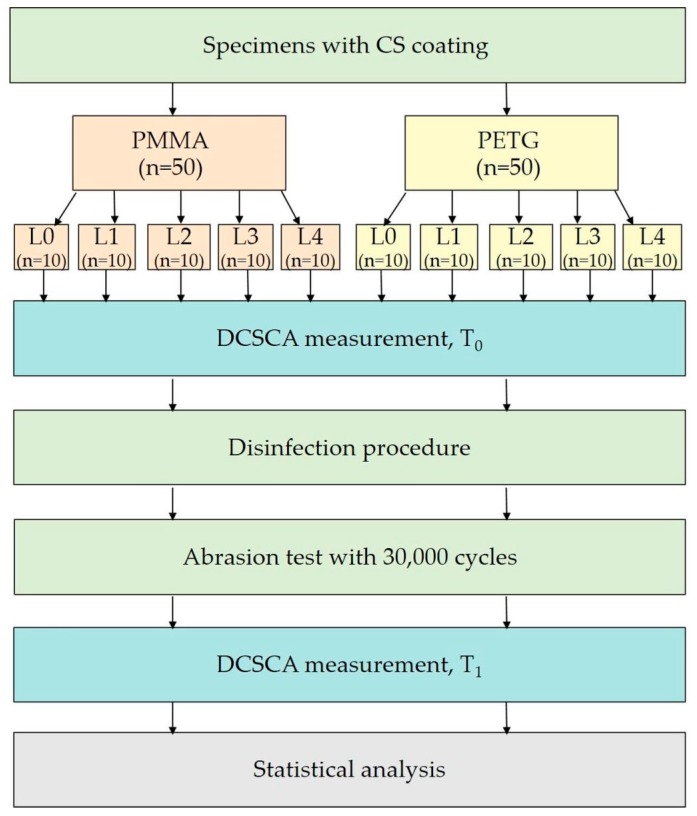
Study design. Baseline measurement (T_0_) of damaged chitosan coating area (DCSCA), measurement after disinfection/abrasion test (T_1_).

To evaluate the abrasion resistance of CS coatings after disinfection procedures, cleaning of the specimens by brushing was simulated. For this abrasion test, all PMMA and PETG specimens were placed into a tooth-brushing simulator (Willytec GmbH, Munich, Germany). Long-term use with 30,000 cycles of linear brushing behavior (load 2 N, 2 cycles/s, 32 °C) with soft brushes (Elmex Sensitive, GABA GmbH, Therwil, Schweiz) in artificial saliva (UKD Pharmacy, Dresden, Germany, Table A2, Appendix A) was simulated. An overview of the study protocol is shown in Figure 5.

### 2.5. Scanning Electron Microscopy (SEM)

The morphology of the CS-coated surfaces after disinfection was additionally evaluated using SEM (XL30 ESEM, Philips Electron Optics, Eindhoven, The Netherlands). One additional specimen per group was prepared in order to produce SEM images: before and after disinfection. The samples were coated with gold and the images were captured at a magnification of 120×, at an accelerating voltage of 20 kV and at a working distance of 7.6 mm.

### 2.6. Statistical Analysis

For the statistical analysis, the damage-score (DS) was computed by DCSCA at T_1_ as a percentage of the difference between the total specimen surface (127.7 mm^2^) and DCSCA at T_0_, as shown in Equation (1):

DS = [100 × (T_1_ − T_0_)]/(127.7 − T_0_) (%) (1)

where T_0_ is the DCSCA measurement at the baseline and T_1_ after disinfection/abrasion test.

An Analysis of Variance (ANOVA) was conducted using a linear model to determine the effects of the disinfectant (L0–L4), the specimen material (PETG, PMMA), and their interaction on DS. To establish homoscedasticity, a log-transformation was applied on DS. Then, the model was fitted using an M-estimator [40] to reduce the effects of potential outliers on the observed data. Tukey post-hoc comparisons were conducted for all pairs of tested disinfectants within each material group, and for both materials within each disinfectant group. *p*-Values < 0.05 were considered to indicate statistical significant differences. For statistical analysis, R software (R Core Team 2016, R Foundation for Statistical Computing, Vienna, Austria) was used.

## 3. Results

The ANOVA revealed a significant main effect related to the disinfectant (F(4,90) = 48.45, *p* < 0.001) as well as a significant interaction between disinfectant and material (F(4,90) = 14.85, *p* < 0.001).

Furthermore, post-hoc tests revealed that of all the disinfectants tested L2 showed the lowest DS for PETG (1.2% ± 2%) and PMMA (9.3% ± 5.8%).

For PETG, L2 showed significantly lower DS than the other tested disinfectants, including the control group (L0 *z* = 7.36, *p* < 0.001; L1 *z* = 8.96, *p* < 0.001; L3 *z* = −12.72, *p* < 0.001, and L4 *z* = −11.14, *p* < 0.001). Also for PMMA material, L2 disinfectant demonstrated significantly lower DS compared to disinfectants L3 (*z* = −3.31, *p* = 0.02), and L4 (*z* = −4.49, *p* < 0.001) but similar to the control group.

The highest DS was noted for disinfectants L3 and L4 for PETG material (60% ± 15% and 41.8% ± 24.8%).

Comparing both control groups, the DS for PMMA (5.7% ± 4.8%) was significantly smaller than for PETG (15.4% ± 10%) when tested with artificial saliva (*z* = 3.7, *p* = 0.005). Although the L2 caused the smallest alteration to the CS coating on PETG (1.2% ± 2%) and PMMA (9.3% ± 5.8%), the alteration on PMMA was significantly higher than on PETG (*z* = −5.74, *p* < 0.001).

**Figure 6:**
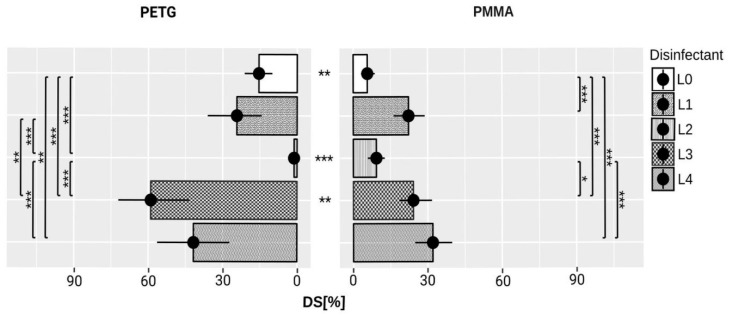
Diagram shows damage scores (DS) in tested disinfectant groups (L1–L4) and control groups (L0). Significant differences between disinfectants and materials are indicated with: * *p* < 0.05, ** *p* < 0.01, and *** *p* < 0.001.

The means and standard deviations of DCSCA at T_0_, and T_1_ are shown in Table A3, (Appendix A); the data for DS are shown in Figure 6.

**Figure 7:**
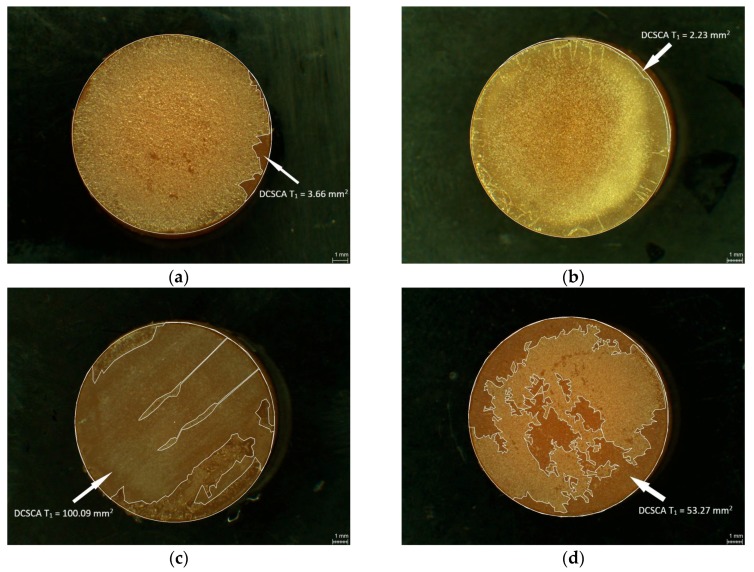
Selected specimens showing damaged CS coating after disinfection/abrasion tests (DCSCA at T_1_ are marked with an arrow): PMMA control group (**a**); PETG L2 (**b**); PETG L3 (**c**); PMMA L4 (**d**).

Figure 7a–d shows damaged CS coating on PMMA and PETG surfaces after disinfection procedures and abrasion tests.

**Figure 8:**
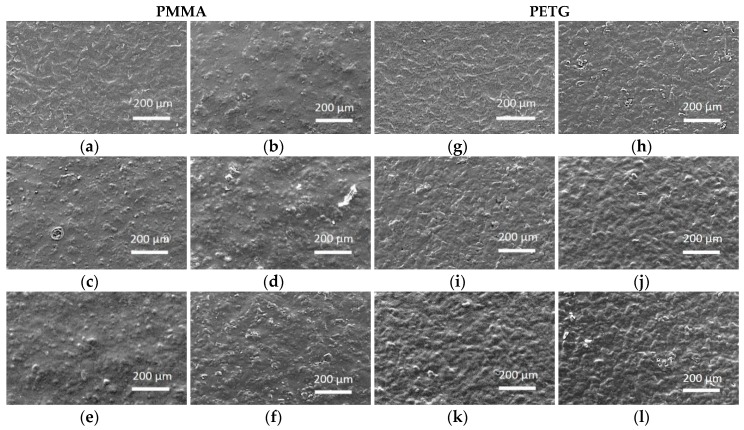
Scanning electron microscopy (SEM) images showing CS coating: PMMA after coating (**a**); PMMA control group L0 (**b**); PMMA L1 (**c**); PMMA L2 (**d**); PMMA L3 (**e**); PMMA L4 (**f**); PETG after coating (**g**); PETG control group L0 (**h**); PETG L1 (**i**); PETG L2 (**j**); PETG L3 (**k**); PETG L4 (**l**).

Figure 8a–l presents SEM images of the surface of the CS coated specimens both before (Figure 8a,g) and after disinfection (Figure 8c–f,i–l). Irrespective of disinfection type, slightly surface morphology changes were observed, without noticeable damages after disinfection.

## 4. Discussion

This study investigated the influence of chemical disinfection of CS coated PMMA and PETG materials in order to pursue international recommendations and to reduce cross contamination risks [24,25,26]. All three stated null hypotheses had to be fully or partly rejected.

Chemical disinfectants used in this study influenced the CS coating significantly in terms of abrasion resistance. There were also differences in the abrasion resistance of CS coating after disinfection in different types of disinfectants.

The disinfectants with active oxygen such as Silosept and Dentavon contain MPS as an active agent and produce hydroxyl and sulfate radicals. Both free radicals can alter the CS coating, probably due to depolymerization reactions and oxidative CS chain scission [41,42,43,44]. As shown in previous studies, the main route in the depolymerization of polymers is scission of the glycosidic bonds in the polymer chain [41]. The oxidative free radical degeneration of CS is initiated by hydroxyl and/or sulfate radicals in aqueous solutions [41]. The cationic amino group on the C-2 carbon of the CS electrostatically attracts the anionic sulfate radicals which can then attack the C-4 carbon in the CS and subtract the hydrogen from it by transporting the radical to it [42]. This could result in breaking of the glycosidic bond in the CS main chain [42]. Also, the hydroxyl radicals can cause deamination of CS, as they abstract hydrogen atoms from C-1 and C-2 carbons leading to chain scission [44]. The degradation of CS can be restrained through protonation of amino groups [44]. Otherwise, more exposed amino groups make CS sensitive to chain scission by free hydroxyl radicals [43]. Through the chain scission and degradation, the molecular weight of CS decreases and CS molecules become water soluble [43,45].

These interactions can explain the decreased abrasion resistance of CS coatings after disinfection with disinfectants containing active oxygen (MPS).

In contrast, disinfection with GA seems to stabilize the CS coating, especially on PETG material. After 30,000 abrasion cycles, only about 1% of the initial CS coating was missing. This can be explained as a process of chemical gelation through crosslinking reactions of CS and GA by ethylenic double bonds [46,47]. GA is a well-known chemical crosslinker for many biopolymers [47,48,49]. Two main crosslinking reactions are proposed to explain reactions between CS and GA. One of these describes Schiff-Base reactions; the second Michael reactions [46]. According to Schiff-Base reactions, proteins and polymers react with their functional groups with GA [47]. CS also crosslinks with GA in acetic solutions by forming imine bonds (N=C) stabilized by ethylenic bonds. Through this interaction, the chemical and physical properties of the crosslinked polymer change. With increasing GA concentration, the particle size and the crystallinity of the crosslinked polymer decreases [46]. Nevertheless, the main disadvantage of GA as a crosslinker is its free aldehyde groups. These functional groups are cytotoxic and induce inflammatory reactions [47]. However, the crosslinking of polymers with GA can produce mechanically stable membranes or scaffolds. Hence, different methods for GA detoxification, for example, through rinsing with free amine groups, have been described [47]. As mentioned, the main concern about GA as a crosslinker is its cytotoxicity, depending on the concentration of GA and free aldehyde groups [47,49]. So, beyond the fact that GA is a widely used crosslinker, the possibility of significant cell toxicity and biohazard activity limits its use in biomedical products [47]. For this reason, further studies on the release of GA from CS coating, the degree of crosslinking between GA and CS coating, as well as the biocompatibility of GA modified CS coating are needed and at the moment no clinical recommendation can be postulated.

Apart from GA, QUATs are also a component of the MD 520 disinfectant. As shown in previous studies, CS can also interact with QUATs by covalent bonds [50,51]. Quaternary ammonium salts are often used for synthesis of water-soluble quaternized CS derivatives in the presence of e.g., aldehydes [51]. Alkyl groups can be introduced into the amine groups of CS by forming Schiff’s Base intermediates and so *N*-alkyl CS derivatives can be prepared, which can be later quaternized [51].

Also, interactions between CS and alkyl amines and the formation of *N*-(aminoalkyl) CS derivatives have been described. These can be used in drug delivery by forming microspheres [50]. However, on reviewing the literature, the authors did not find studies describing the effects of alkyl amines and quaternary ammonium groups on CS membranes or coatings. Possible interactions between these compounds and a decreased abrasion resistance of CS coating after disinfection with disinfectants containing QUATs and alkyl amine can be summarized. Further studies are needed to understand the interaction mechanisms of these disinfectants on CS coatings.

Besides interaction with the different components of the disinfectants, disinfectant pH value can also influence the CS coating. CS behaves as a weak polybase and its solubility is pH sensitive. CS dissolves easily at a pH below 6.5 [52]. CS salts such as CS acetate also demonstrate pH-dependent solubility and lose their positive charge and precipitate at neutral pH [53]. CS films from CS dissolved in diluted inorganic or organic acids are soluble in water or acidic medium; however, neutralization can improve the stability of CS films [54]. Kam et al. showed that neutralized CS acetate film is relatively insoluble in water [55]. The rudimental solubility of neutralized chitosan acetate film in water can be explained on the basis of residues of acetic acid in the CS film [55].

Apart from Printosept-ID which has a basic pH (containing QUATs and alkyl amines), all the other disinfectants tested have acidic pH values above 4. The reaction with GA is more favorable at basic and neutral pH but, as described by Li et al., it is also possible under acidic pH [46]. This acidic pH can trigger the crosslinking reaction and also contribute to good abrasion stability of CS coating disinfected in disinfectants containing GA. Under neutral and basic pH, CS is not soluble. The disinfectant with mixed QUATs and alkyl amines (Printosept-ID) has a basic pH which could also maintain relatively good abrasion stability of disinfected CS coatings.

Our previous studies describe reliable methods to coat PMMA and PETG with CS [19,20]. Both 2% and 4% CS acetate solutions can be used for CS coating [20]. In this study, 2% CS acetate solution was used, because of its better applicability [20]. Through silica coating of PMMA and PETG, their surfaces are enriched with silica and achieve a hydrophilic character [20]. Furthermore, it has be shown that the wettability of the surface is the key factor when coating with CS [20]. The presence of silica in the specimen surface and so its increased wettability before coating is stated to be a key factor to bond CS to PMMA and PETG [20]. Previous studies have shown a physical association between glucopyranose rings of CS and silica or silicate, probably through dipole–dipole and hydrogen-bonding interactions [56].

In our previous study, the remaining CS coating for PETG after abrasion tests was above 95% [20]. This finding is contrary to our present study where the remaining CS coating was about 85%. In the present study, differences in abrasion resistance of CS coating on PMMA and PETG were also shown. In contrast to our previous study, the CS coating on PMMA was more abrasion resistant compared to CS coating on PETG [20]. Nevertheless, in contrast to the present study, our previous study did not include the immersion of the specimens in distilled water before abrasion tests [20]. The CS coating on PETG seems to be more sensitive to water storage than PMMA. The CS binding to PETG seems to be more hydrolysis sensitive. Apart from this, the resistance of CS coating on PETG after disinfection with disinfectants containing GA/QUATs was excellent. GA crosslinking on PETG seems to be more effective than on PMMA. Nevertheless, the CS coating loss of less than 10% for PMMA is clinically negligible. These aspects and chemical interactions need further studies before first application of tests in a clinical environment.

For this and our previous studies, CS with DDA of 87.6–92.5% and MW of 200–400 kDa was used. High DDA was chosen as the antimicrobial activity of CS and its solubility in acids increased with increasing DDA [12,57]. Moreover, CS with high DDA has a greater hemostatic effect; nevertheless, it is possible for it to deform the erythrocytes [58]. For hemostatic CS bandages, the most preferable DDA is described as being between 85% and 95% [59]. CS with MW of 200–400 kDa was chosen because the MMW CS and HMW CS show an equal or even greater effect against bacteria compared to LMW, and MW of about 300 kDA is described as being the most preferable for hemostatic bandages [12,59]. Moreover, in terms of antifungal activity, HMW CS has been shown to decrease the pathogenicity of *C. albicans* for treating oral candidiasis [60]. However, other studies show that LMW is more effective against fungi [12]. Further studies with LMW CS could also be interesting when using CS coating in treatment of denture stomatitis, as it is closely associated with colonization by *C. albicans*. Nevertheless, the disadvantage of LMW is its faster degradation than is the case for HMW [61]. This could affect the effectiveness and longevity of the CS coating.

The qualitative assessment of the specimen surfaces under SEM after disinfection did not reveal noticeable damages in surface morphology. For the measurement of DCSCA, digital planimetry was chosen, as the CS coating would have been affected and/or potentially destroyed by the electron beam under SEM analysis.

Nevertheless, a limitation of this study is that no infrared (IR)-analysis or X-ray photoelectron spectroscopy (XPS) was conducted. This aspect needs further analysis. XPS analyzes of neutralized chitosan acetate film by other authors suggest the presence of hydrated crystals and crystals of α-chitin chain segments [55].

A further limitation of this study is that no antimicrobial and hemostatic tests have been conducted. These activities have been established in many other studies [1,5,6,7,8,9,10,11,12].

The aim of this study was primarily to evaluate the abrasion stability of the CS coating after disinfection, leading to further in vitro and in vivo tests if a sufficient disinfection/sterilization method has been established.

Abrasion tests with 30,000 brushing cycles simulate the long-term use of dental appliances and can be considered a worst-case scenario. About 1000 brushing cycles simulate about one month of use and brushing of 30,000 cycles simulates about 2 years [62,63]. A treatment for denture stomatitis can take up to 3 months, the appliances for bleeding control up to 1 week. As shown in the present study, after simulating for 2 years in most cases a third to a half of the CS coating was present on the specimens. Further studies simulating shorter periods of use, with lower numbers of brushing cycles, but higher numbers of disinfection cycles are necessary, as each time a patient consults a practitioner disinfection could be needed.

As an alternative to disinfection, low temperature sterilization with ethylene oxide (ETO) can be used. Current recommendations for sterilization of polymer-based implantable medical devices from PMMA are ETO or hydrogen peroxide (H_2_O_2_) and for polyethylene ETO and radiation [27]. ETO is a low-temperature sterilization method, widely used in healthcare for years. Alkylation is a mode of the biocidal action of ETO, in which saturated hydrocarbon groups are added to reactive amino, sulfhydryl, hydroxyl or carboxyl groups [27]. Its long cycle time, high costs and its potential as to be a biohazard (toxic residues), and human carcinogen are the main disadvantages of ETO [64]. To limit these disadvantages, aeration of sterilized devices is needed and allowable ETO limits are stated [31]. As an alternative to chemical disinfection, ETO could presumably be used for sterilization of dental appliances coated with CS such as dentures and surgical splints. In previous studies, sterilization of CS membranes or coatings with ETO has been studied and recommended, due to its low effect on CS membrane morphology, on the percentage of CS chain breaks, and the lack of effect of CS layer bonding to substrate material [14,15]. Nevertheless, the disadvantages of this sterilization method, including its toxicity, flammability, environmental risks, and possible contamination of the materials with ETO residues, limits the applicability of this method [14]. Moreover, chemical disinfection is recommended as an adequate decontamination method for dentures and prosthodontic materials and sterilization as a higher level of decontamination is not required [26,31].

Although CS has been studied with respect to many biomedical applications, its sterilization and disinfection is still linked to many problems, such as chemical alteration or possible toxic residues [65]. The present study shares this conclusion. Further studies are needed to find an optimal disinfection/sterilization method for CS coated surfaces.

## 5. Conclusions

Although disinfection with disinfectants containing GA and QUATs seems to stabilize the CS coating, the possible free aldehyde groups could act as biohazard. Within the limitations of this study, disinfectants containing QUATs and alkyl amines could be applicable for disinfection of CS coatings, as after simulation of long-term use the remaining CS coating can be seen as acceptable. Active oxygen leads to the greatest alteration to the CS coating, and this effect is material dependent. Further analyses with IR-spectroscopy or XPS and studies on interactions between disinfected CS coatings and human tissue, its biocompatibility, toxicity and degradation, are needed before further applications in clinical trials are justified.

## Figures and Tables

**Table 1:** Information about tested chemical disinfectants.

Brand Name	ID	Manufacturer	Group of Active Agents	Active Agents	pH
Printosept- ID	L1	Alpro Medical, St. Georgen, Germany	QUAT, alkyl amine	*N*,*N*-Didecyl-N-methylpoly(oxyethyl)- ammoniumpropionate, *N*-(3-Aminopropyl)-N-dodecylpropane-1,3- diamine	10.5–11.5
MD 520	L2	Dürr Dental, Bietigheim- Bissingen, Germany	GA, QUAT	Glutardialdehyde, Aalkyl-benzyl-dimethyl-ammonium- chloride	~4.3
Silosept	L3	Kettenbach, Eschenbur, Germany	active oxygen	Pentapotassiumbis(peroxymono-sulphate)- bis(sulphate) (MPS)	3.71 (1% solution)
Dentavon	L4	Schülke, Norderstedt, Germany	active oxygen	Pentapotassiumbis(peroxymono-sulphate)- bis(sulphate) (MPS)	~4

## Appendix A

**Table A1:** Preparation and disinfection times for tested disinfectants.

ID	Brand Name	Batch-No	Dosage	Exposure Time (min)
L1	Printosept-ID	294102	Solution ready to use	5
L2	MD 520	1401207	Solution ready to use	10
L3	Silosept^®^	1241466	20 g of Silosept into 2 L of lukewarm water (for 2% solution)	10
L4	Dentavon^®^	1240192	40 g Dentavon into 2 L of lukewarm water (for 2% solution)	10

**Table A2:** Composition of artificial saliva.

Ingredients	%
Aqua dest.	82.93
Hydroxyethyl cellulose	12.5
Sorbitol solution	4.28
Potassium chloride	0.12
Sodium chloride	0.08
Sodium monohydrogenphosphate 12 H_2_O	0.06
Calcium chloride 2 H_2_O	0.02
Magnesium chloride 6 H_2_O	0.01
Preservative Propyl 4-Hydroxybenzoate	<0.01

**Table A3:** Descriptive data for damaged chitosan coating area (DCSCA) at baseline T_0_ and after disinfection/abrasion test T_1_.

Material	Disinfectant	T_0_ Mean (mm^2^) ± SD	T_1_ Mean (mm^2^) ± SD
	L0	0.8 ± 1.5	7.2 ± 6.1
	L1	3.1 ± 2.3	27.8 ± 13.5
**PMMA**	L2	3.5 ± 4	11.4 ± 6.8
	L3	4.2 ± 2.8	29.9 ± 14.6
	L4	2.6 ± 1.3	40 ± 16.1
	L0	0.2 ± 0.5	19.7 ± 12.6
	L1	0 ± 0	31 ± 24.9
**PETG**	L2	0 ± 0	1.5 ± 2.5
	L3	0.1 ± 0.3	75.3 ± 31.6
	L4	0.4 ± 1	53 ± 31.1
